# Bibliography

**Published:** 1896-05

**Authors:** 


### Bibliography.

Pathologie des Dents et de la Bouche. Par le Dr. Léon Frey, ancien interne des hôpitaux de Paris, Professeur-supp. chargé du cours de Pathologie spéciale à l'École dentaire de Paris. Paris: Librairie, J. B. Baillière et Fils, 1896.

This manual of two hundred and seventy-four pages and thirty-two illustrations covers more of dental pathology than is frequently found in works of greater pretentions; indeed, for the purpose intended, it is an admirable condensation which might be profitably imitated by those attempting similar publications in this country. With some additions in harmony with the teachings in this part of the world, it would be an excellent work to translate for the use of students.

A History of the Chronic Degenerative Diseases of the Central Nervous System. By Thomas Kirkpatrick Monro, M.A., M.D., Fellow of the Faculty of Physicians and Surgeons of Glasgow, etc. Glasgow: Alexander Macdougal, 68 Mitchell Street, 1895.

This is a series of essays constituting "historical studies of those chronic disorders which depend upon primary degenerative changes in the structure of the central nervous system."

The table of contents will indicate its value to those interested in these special studies. "History of tabes; history of primary spastic paralysis; of ataxic paraplegia; of hereditary ataxy; of progressive muscular atrophy; of bulbar paralysis; of ophthalmoplegia; of the peroneal type of muscular atrophy; of disseminated sclerosis."

As this list indicates, the eighty-seven pages of which this volume is composed covers matters of importance in the study of neurology, and will be of special value to writers who may desire to be correct in preparing the history of the various degenerative diseases upon which the author treats.

